# Extension of the minimal functional unit of the RNA polymerase II CTD from yeast to mammalian cells

**DOI:** 10.1098/rsbl.2019.0068

**Published:** 2019-05-15

**Authors:** Nilay Shah, Tim-Michael Decker, Dirk Eick

**Affiliations:** 1 Department of Molecular Epigenetics, Helmholtz Center Munich, Center for Integrated Protein Science Munich (CIPSM), Marchioninistrasse 25, 81377 Munich, Germany; 2 Department of Biochemistry, University of Colorado, Boulder, CO 80303, USA

**Keywords:** RNA polymerase II (Pol II), C-terminal domain (CTD), heptad-repeats, low-complexity domain, minimal functional unit (MFU)

## Abstract

The carboxy-terminal domain (CTD) of the largest subunit of RNA polymerase II (Pol II) consists of 26 and 52 heptad-repeats in yeast and mammals, respectively. Studies in yeast showed that the strong periodicity of the YSPTSPS heptads is dispensable for cell growth and that di-heptads interspersed by spacers can act as minimal functional units (MFUs) to fulfil all essential CTD functions. Here, we show that the MFU of mammalian cells is significantly larger than in yeast and consists of penta-heptads. We further show that the distance between two MFUs is critical for the functions of mammalian CTD. Our study suggests that the general structure of the CTD remained largely unchanged in yeast and mammals; however, besides the number of heptad-repeats, also the length of the MFU significantly increased in mammals.

## 1. Introduction

The carboxy-terminal domain (CTD) of Pol II is a repetitive low-complexity domain that extends from the large subunit (Rpb1) in eukaryotes. The CTD comprises heptad-repeats with the consensus sequence of Tyr_1_-Ser_2_-Pro_3_-Thr_4_-Ser_5_-Pro_6_-Ser_7_. The general structure of the CTD remains largely unchanged in yeasts and metazoans; however, the number of repeats varies remarkably. There are 26 repeats in the CTD of *Saccharomyces cerevisiae* [1], 29 in *Schizosaccharomyces pombe* [2] and 52 in mammals [3]. The CTD serves as a binding platform for the recruitment of protein complexes and helps to coordinate the entire transcription cycle, RNA maturation and epigenetically modifying the chromatin [4–7]. Deletion of the entire CTD is lethal in yeast, *Drosophila* and mammalian cells [8–11].

Genetic studies in yeast showed that the strong periodicity of heptad-repeats is not an absolute requirement for viability. Insertion of a single alanine residue between adjacent repeats is lethal, while insertion of alanine residues between pairs of heptads is well tolerated both in *S. cerevisiae* [12] and in *S. pombe* [13]. These analyses led to the concept of the minimal functional unit (MFU), in which essential CTD functions are accomplished through the interaction of protein factors with motif(s) that lie within di-heptads. Further analysis in *S. cerevisiae* revealed that the substitution of ‘SPS’ at positions 5–7 of the distal repeat with alanine (YSPTSPS YSPTAAA) or the removal of these residues (YSPTSPS YSPS) had no discernible impact on cell growth*.* These mutants were termed ‘AR’ and ‘252’, respectively [14]. Similar observations were made in *S. pombe*, where a mutant with an array of ‘YSPTSPS YSPAAA’, grew well at 18–34°C, but did not thrive at 37°C [13]. Together, these experiments suggested that a decapeptide unit could serve as an MFU and arrays of this MFU could suffice for the CTD functions in yeast.

Here, we identified the MFU in the CTD of mammalian cells by taking advantage of an Rpb1 knockin–knockout system, using an Rpb1 mutant that confers resistance to α-amanitin [10,11]. Our experiments demonstrate that (i) the MFU of mammalian CTD is larger than in yeast and consists of penta-heptads; and (ii) similar to yeast, the distance between two MFUs is critical for mammalian cell growth.

## 2. Material and methods

### (a) Establishing stable cell lines

CTD sequences of rWT and CTD mutants (mAR, m252, Con 48, 15_AP, 8_AP, 6_AP, 5_AP, 4_AP, 3_AP, 2_AP, 6_2AP and 5_2AP) were synthesized by GeneArt (Regensburg, Germany) using human codon optimization. These constructs were cloned into LS*mock vector [11] and sequenced before transfection. 20 × 10^6^ Raji cells were used to transfect full-length Rpb1 vector carrying the rWT or CTD constructs using electroporation (10 µg of plasmid, 960 µF and 250 V). Polyclonal cell lines were established by selection with G418 (1 mg ml^−1^) for two to three weeks. Once the cell viability reached approximately 95%, the expression of recombinant Pol II was induced by removing tetracycline by washing the cells three times with 50 ml PBS supplemented with 1% fetal calf serum (FCS, Gibco). Twenty-four hours after induction, 2 µg ml^−1^ of α-amanitin (Sigma) was added to inhibit endogenous Pol II and cell lines were maintained in RPMI 1640 complete medium (10% FCS, 100 U ml^−1^ penicillin, 100 μg ml^−1^ streptomycin) at 37°C and 5% CO_2_.

### (b) Antibodies

Monoclonal antibodies specific for haemagglutinin (HA)-tag (3F10, Roche) and α-tubulin (T6199, Sigma) are commercially available. Monoclonal antibodies against Rpb1 (Pol 3.3), Ser2P (3E10), Ser5P (3E8), Ser7P (4E12), Tyr1P (3D12) and Thr4P (6D7) were described previously [15–17].

### (c) Western blot analysis

Cells were washed twice with PBS and lysed in 2× Laemmli buffer. Proteins were separated by SDS-PAGE and transferred to a nitrocellulose membrane (GE Healthcare). The membranes were first incubated with 5% milk in TBS-T for 1 h to block unspecific binding of antibodies. Membranes were then incubated with the primary antibody in blocking solution at 4°C overnight. Next day, the membranes were washed with 1× TBS-T and incubated for 1 h at room temperature (RT) with horseradish peroxidase (HRP)-conjugated secondary antibodies against rat (Sigma) or mouse (Promega) to be detected by chemoluminescence or incubated for 90 min at RT with IRDye-labelled, secondary antibodies against rat (680 nm; Alexa, Invitrogen) and mouse (800 nm; Rockford, Biomol) and analysed using an Odyssey Imaging System (Li-Cor).

### (d) Growth kinetics

20 × 10^6^ cells of each cell line were induced and the numbers of living cells (*N*_L_) and dead cells (*N*_D_) were counted every day using trypan blue staining. The percentage of cell viability (*N*_V_) was calculated using the following formula *N*_V_ = (*N*_L_/*N*_L_ + *N*_D_) × 100. To calculate the cumulative living cell number, the total number of living cells (*N*_L_) was multiplied by the factor by which the cell culture was split.

### (e) Statistical analysis

Two clones from each mutant were transfected into Raji cells to generate two biological replicates. Following the induction, three individual measurements of living cells and dead cells from each replicate were taken every day using a Fuchs–Rosenthal chamber. The mean of these three measurements for each biological replicate was calculated. Following this, the s.d. of the mean was used to calculate s.e.m. and plotted in the graphs.

## 3. Results and discussion

### (a) The MFU of the CTD is different in yeast and mammalian cells

**Figure 1.**
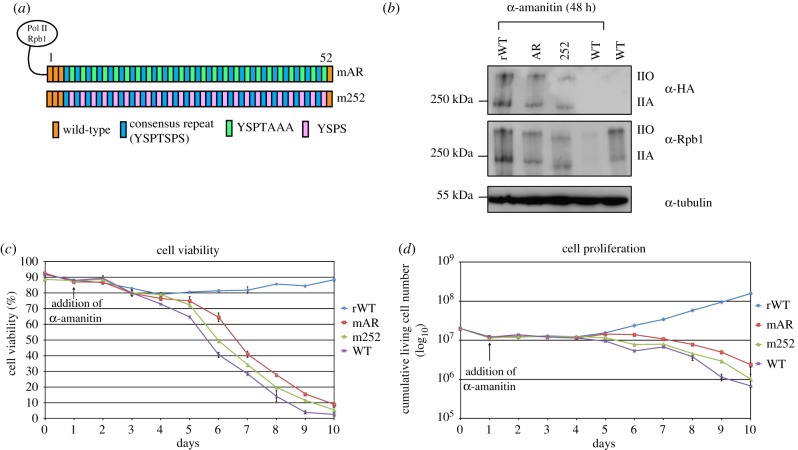
(*a*) Schematic representation of CTD mutants mAR and m252. Each box represents an individual CTD repeat. Wild-type (WT) repeats (orange), consensus repeats (blue), YSPTAAA (green) and YSPS (pink) repeats are depicted. (*b*) Western blot analysis showing the expression of recombinant Pol II in mutants after 48 h of α-amanitin treatment (72 h of induction) using α-HA (3F10) antibody. rWT cells were used as a positive control. Expression of total Pol II (endogenous and recombinant) in CTD mutants was detected using α-Rpb1 (Pol 3.3) antibody. (*c*,*d*) Graphs representing the percentage of cell viability (*c*) and cell proliferation *(d)* in rWT, CTD mutants and WT-Raji cells, for a period of 10 days. The numbers of living cells (*N*_L_) and dead cells (*N*_D_) were counted every day using trypan blue stain (*n* = 2).

Previous studies in *S. cerevisiae* and *S. pombe* identified a decapeptide unit of ‘YSPTSPSYSPS’ as an irreducible MFU of the CTD. We tested if CTD mutants with arrays of this decapeptide unit can suffice for cell proliferation of mammalian cells. We designed two CTD constructs, mAR and m252 (figure 1*a*), similar to the CTD mutants AR and 252 described in *S. cerevisiae* [14]. Stable cell lines expressing the CTD mutant were generated using an Rpb1 knockin–knockout system. Western blot analysis showed that the recombinant wild-type Pol II CTD (rWT) and the two CTD mutants expressed the hypophosphorylated (IIA) and hyperphosphorylated (IIO) forms of Rpb1 (figure 1*b*). This suggests that the mutants are transcriptionally active. Next, we monitored the growth kinetics of mutants by calculating the numbers of living and dead cells for a period of 10 days. Both mutants showed a continuous decline in the percentage of cell viability (figure 1*c*) and cumulative living cell number (figure 1*d*). Both mutants had less than 10% of living cells after 10 days of α-amanitin selection and eventually displayed a lethal phenotype. Together, these experiments showed that arrays of MFUs that confer growth and viability in yeast could not confer viability in mammalian cells. Thus, we conclude that the MFU of the CTD in mammalian cells is different from yeast.

### (b) MFU of mammalian CTD consists of penta-heptads

**Figure 2.**
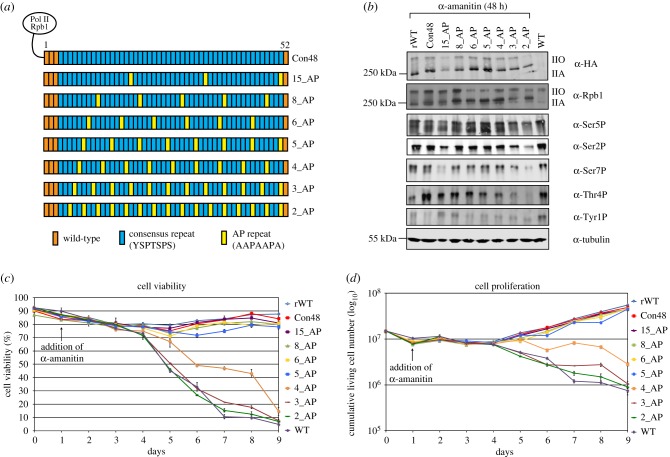
(*a*) Schematic representation of CTD mutants with one AP spacers between arrays of MFUs. (*b*) Western blot analysis showing the expression of recombinant Pol II, total Pol II, Ser2P (3E10), Ser5P (3E8), Ser7P (4E12), Thr4P (6D7) and Tyr1P (3E12) in rWT, CTD mutants and WT-Raji cells. (*c*,*d*) Graphs showing the percentage of cell viability (*c*) and cell proliferation (*d*) in these cells (*n* = 2).

We next determined the MFU of mammalian CTD. Repeats 1–3 and repeat 52 are important for the stability of CTD in mammalian cells, as the deletion of these repeats leads to the cleavage and degradation of the CTD from Pol II [18,19] (figure 2*a*). The mutant (Con48) in which repeats 4–51 of wild-type CTD were replaced by consensus heptads showed wild-type-like cell growth and proliferation, demonstrating that the non-consensus repeats are dispensable for cell growth [19]. Taking this information, we designed a set of CTD mutants using Con48 as template mutant. We replaced a consensus heptad with an AP spacer (AAPAAPA) after every 15, 8, 6, 5, 4, 3 or 2 consensus heptads (figure 2*a*). Previous work in yeast showed that the replacement of proline by alanine in spacers is lethal [20] and thus we conserved prolines at their canonical positions. This strategy allowed us to design a set of CTD mutants in which the size of the MFU varies between 15 and 2 consensus heptads (figure 2*a*). Western blot analysis revealed that all mutants express the IIA and IIO forms of the Rpb1 (figure 2*b*). Furthermore, we detected comparable levels of CTD phosphorylation in all mutants, suggesting that all mutants are transcriptionally active and elongation competent. In growth kinetics, rWT and the mutant Con48 showed similar cell viability and proliferation (figure 2*c*,*d*). CTD mutants with arrays of MFUs of five or more consensus heptads (15_AP to 5_AP) were also viable under α-amanitin selection. Mutants 6_AP and 5_AP had a slight reduction in the percentage of cell viability as compared with Con48 but had nearly 80% cell viability after 9 days of selection, suggesting a viable phenotype. Interestingly, CTD mutants with arrays of MFUs of four or less consensus heptads showed a continuous decline in the percentage of cell viability and cumulative living cell numbers. The cell viability reached less than 10% after 9 days of α-amanitin selection, indicating a lethal phenotype for these mutants without any indication for growth adaptation. Together, we conclude that the MFU of mammalian CTD is significantly larger than in yeast and lies within penta-heptads.

Our results suggest that, similar to yeast, the strong periodicity of heptad-repeats is not an absolute requirement in mammalian cells. However, in mammalian cells, essential CTD functions are accomplished through a motif that lies within penta-heptads in contrast to di-heptads in yeast [12]. Does this mean that the 35 amino acids covered by penta-heptads are all required for the functionality of mammalian MFUs? Previous studies have designed CTD variants to map the phosphorylation sites along the entire CTD in yeast [21] and mammals [22]. In these studies, a number of mutations including lysine (K) or arginine (R) were introduced after every two or three heptads to make the entire CTD accessible to mass spectrometry. Interestingly, these CTD variants were viable and showed comparable cell proliferation to that of recombinant wild-type CTD. This demonstrates that even within an MFU, mutations at certain positions are tolerated, suggesting that not all residues in MFUs of penta-heptads may be critical for CTD functions in mammalian cells. This is consistent with yeast mutants showing that only the presence of three SP (serine-proline) motifs and two tyrosine residues spaced seven amino acids apart were an absolute requirement for an MFU [14]. This appears also likely for mammalian CTD. However, the question of critical amino acids within a mammalian MFU has not been addressed in this study.

Why is the MFU of mammalian CTD longer than in yeast? It could well be that during evolution the CTD became more versatile to recruit larger proteins or protein complexes to regulate transcription-coupled processes. Additionally, CTD is an intrinsically disordered low-complexity domain that can undergo liquid–liquid phase separation *in vitro* and could cluster Pol II in living cells [23]. CTD interacts with other low-complexity proteins, including, MED-1, a subunit of the Mediator complex [24,25] to regulate Pol II localization and gene expression. The head and the middle module of Mediator make multiple contacts with the CTD [26,27] in a tyrosine-1-dependent manner [17]. X-ray crystallography revealed that the head module of Mediator binds a peptide with several heptads [27]. The number of heptad-repeats binding to mammalian Mediator has not been studied; however, unpublished data of our laboratory indicate that a CTD with MFUs containing fewer than five heptad-repeats does not bind Mediator. Finally, mammalian CTD can recruit proteins which are specific for metazoans and not present in yeast. For example, a 14-subunit protein complex called Integrator interacts with the Pol II CTD in a Ser2 and Ser7 phosphorylation-dependent manner [28–30]. Recruitment of Integrator to Pol II CTD is crucial to regulate the 3′-end processing of small nuclear RNA (snRNA) genes [31] as well as polymerase pause–release [32,33]. Thus, mammalian CTD requires a longer MFU motif, probably to accommodate more and/or larger proteins for transcription-coupled processes.

### (c) Spacing between functional units

The distance between MFUs is critical in *S. cerevisiae*, as the growth rate slows down with the increase in the length of the spacer [12]. To understand the significance of distance between MFUs in mammalian cells, we designed CTD mutants in which MFUs of hexa- or penta-heptads were separated by two AP spacers. These mutants were termed as 6_2AP and 5_2AP, respectively (electronic supplementary material, figure S1*a*). Both mutants expressed the IIA and IIO forms of polymerase and showed comparable levels of Ser5 and Ser2 phosphorylation (electronic supplementary material, figure S1*b*). However, both mutants displayed a strongly reduced cell viability of less than 20% after 12 days under α-amanitin selection (electronic supplementary material, figure S1*c,d*). Together, these experiments suggest that the distance between two MFUs is also critical in mammalian cells and that increasing the distance between two MFUs by more than one AP spacer has a severe impact on cell growth. Separating MFUs by two AP spacers may affect the recruitment of certain essential transcription factors or affect the CTD-driven aggregation of Pol II [34], altering gene expression.

## 4. Conclusion

The length of the CTD considerably increased during the evolution from yeast to mammals, but the general structure of the CTD remained largely unchanged. We showed that besides the CTD length, the demand of the MFU length also increased in mammalian cells. This increase in the MFU length probably provides more versatility to the mammalian CTD to coordinate the complex transcription-coupled processes.

## Supplementary Material

Electronic Supplementary Figure S1

## Data accessibility

The data generated during this study are included in this article as figures and as electronic supplementary material. Plasmids harbouring the CTD mutants are available from the corresponding author upon request.
