# Supplementary material for: Extension of the minimal functional unit of the RNA polymerase II CTD from yeast to mammalian cells
Source: Biol Lett. 2019 May 15;15(5):20190068. doi: 10.1098/rsbl.2019.0068 (PMC6548728; doi:10.1098/rsbl.2019.0068)
Supplement: Electronic Supplementary Figure S1 [file rsbl20190068supp1.pdf]

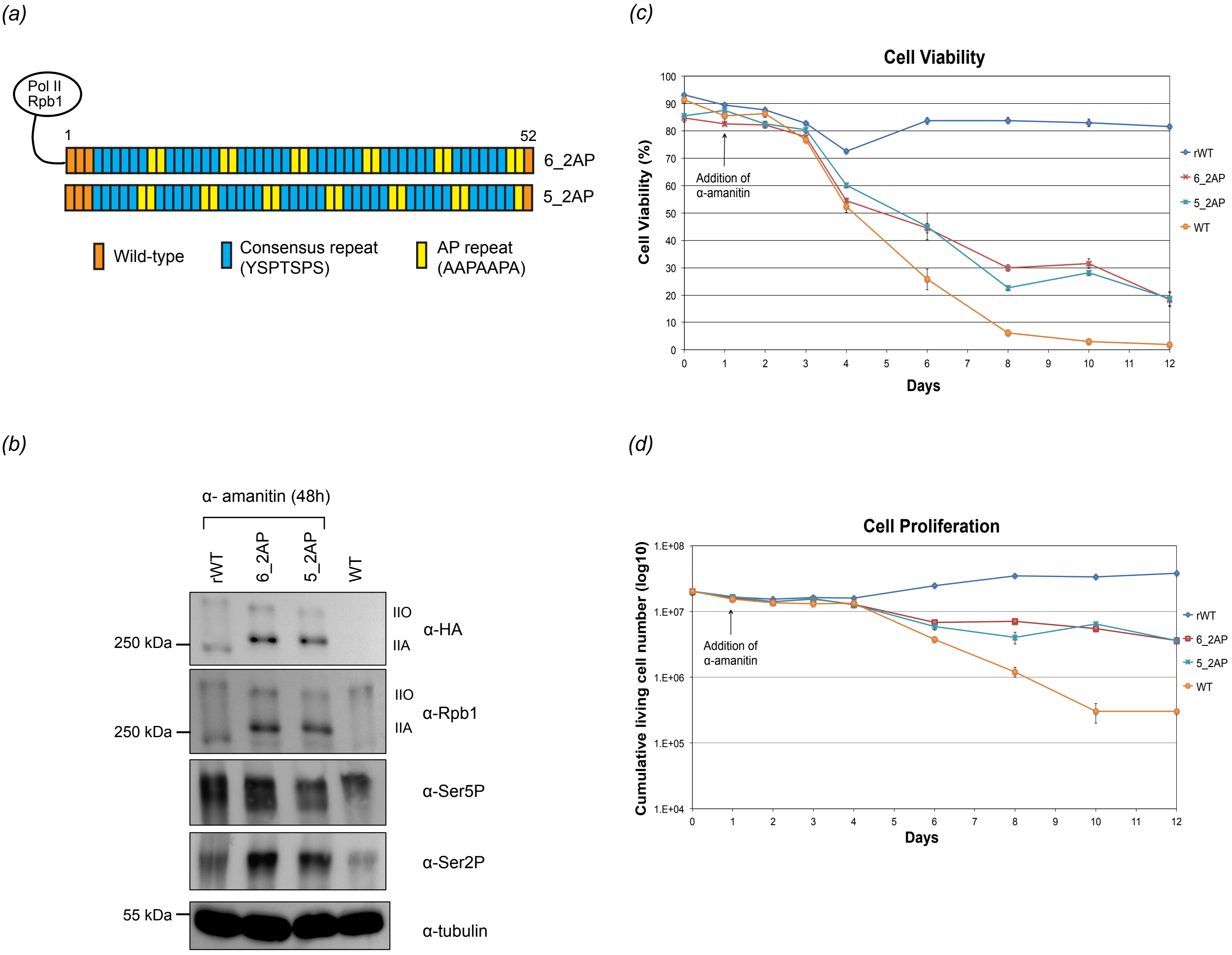

Supplementary Figure S1. (*a*) Schematic representation of CTD mutants with two AP-spacers between arrays of MFUs. (*b*) Western blot analysis and graphs representing the percentage of cell viability (*c*) and cell proliferation (*d*) as described in figure legends 1 and 2 (n = 2).

Electronic Supplementary Figure S1

Manuscript Title: Extension of the Minimal Functional Unit of the RNA Polymerase II CTD from yeast to mammalian cells

Authors: Nilay Shah, Tim-Michael Decker and Dirk Eick
